# Phrenic nerve block with ultrasound-guidance for treatment of hiccups: a case report

**DOI:** 10.1186/1752-1947-5-493

**Published:** 2011-10-03

**Authors:** Kristiina Kuusniemi, Ville Pyylampi

**Affiliations:** 1 Department of Anaesthesiology, Intensive Care, Emergency Care and Pain Medicine, Turku University Hospital, Luolavuorentie 2, Turku 20700, FI-20520, Finland

## Abstract

**Introduction:**

Persistent hiccups can be more than a simple and short-lived nuisance and therefore sometimes call for serious consideration. Hiccupping episodes that last only a few minutes may be annoying, but persistent hiccups may initiate many major complications.

**Case presentation:**

A 72-year-old Caucasian man with spinal stenosis presented for L4-5 laminectomy under spinal anesthesia. The surgery and anesthesia, as well as the perioperative period, passed without any incident, except for persistent postoperative hiccups not responding to conservative and pharmacological treatment. Hiccups resulted in a prolonged hospital stay as they lasted until the seventh postoperative day. On that day, a right-sided ultrasound-guided phrenic nerve block with 5 ml of bupivacaine 5 mg/ml with epinephrine was performed successfully with a single-injection technique. Ten minutes after the procedure the hiccups vanished and a partial sensomotoric block of his right shoulder developed. No adverse effect occurred; our patient could be discharged on the same day and the hiccups did not return.

**Conclusion:**

Ultrasound provides us with non-invasive information regarding anatomy and allows anesthesiologists to visualize needle insertion, to identify the exact location of the injected solution and to avoid such structures as arteries or veins. As such, this method should be actively utilized. In cases where both pharmacological and non-pharmacological treatments prove to be ineffective when treating persistent hiccups, a single-shot ultrasound-guided technique should be considered before the patient becomes exhausted.

## Introduction

Hiccups, or singultus, are caused by synchronous contractions of the diaphragmatic and intercostal muscles followed by the closure of the glottis. Persistent hiccups can be more than a simple short-lived and benign nuisance and therefore sometimes call for serious consideration. Hiccupping episodes that last only a few minutes may be annoying, but persistent hiccups may cause such major complications as dehydration, weight loss, exhaustion, insomnia, delirium, depression, cardiac arrhythmias, electrocardiogram (ECG) artefacts, wound dehiscence, severe reflux esophagitis, and others [1].

Unlike reflexes, hiccups do not appear to have any known protective function. Both pharmacological and non-pharmacological treatments have been used when trying to cure hiccups. Several therapies aiming at disrupting the transmission of impulses along the phrenic nerves have been suggested, a nerve block being one of the methods. On the other hand, persistent hiccups after attempted interscalene brachial plexus block have also been reported [2]. During the past few years, ultrasound (US) has been found useful for anesthetizing and assessing disorders affecting peripheral nerves. Recently a review of the phrenic nerve sonoanatomy has been published, demonstrating a precise mapping of the course of the phrenic nerve in the cervical region. It also calls for clinical applications, such as facilitating the injection of anesthetics when a selective nerve block is considered [3]. In order to cure intractable hiccups, US-guidance has also been used to localize the phrenic nerve for radiotherapy [4]. There is also a case report of an US-guided phrenic nerve catheter placement and a continuous blockade for treating difficult postoperative hiccups [5].

### Case presentation

A 72-year-old Caucasian man (height 171 cm; weight 83 kg) had a medical history of coronary artery disease, hypertension, atrial fibrillation and bilateral hip arthrosis. He was scheduled for L4-5 laminectomy to treat spinal stenosis. The operation was carried out in prone position under spinal anesthesia with bupivacaine 10 mg and clonidine 30 μg in a 4 ml volume diluted with sterile aqua. The surgery lasted 156 min. There were no adverse effects or complications during the surgery or the anesthesia. From the first night following the operation, however, our patient suffered from hiccups. He was treated in all with 50 mg diazepam, 165 mg oxycodone, 2.25 mg dehydrobenzperidol, 20 mg metoclopramide and 100 mg chlorpromazine during the perioperative period. The hiccups continued despite drug therapies and conservative methods (for example drinking water from the opposite side of a glass, paper bag breathing, swallowing dry granulated sugar). According to the radiologist, a chest radiograph revealed a high right-sided diaphragm and there were slight basal atelectases bilaterally but otherwise the chest radiograph was normal. During this hospital visit our patient did not need a central venous line or other procedures on the right side of his neck or shoulder. However, he had had a cannula inserted in his internal jugular vein eight years prior to this visit during an uneventful open heart surgery.

On the seventh postoperative day a right-sided US-guided phrenic nerve block was performed using linear high frequency transducer (S-Nerve, SonoSite Inc., USA) and a 22 G × 50 mm needle (Pajunk UniPlex NanoLine^® ^, Pajunk Medical Produkte GmbH, Germany). The right phrenic nerve was located slightly below the cricoid level where it was separate from the C5, C6 and C7 nerve roots. After aspiration, 5 mL of bupivacaine 5 mg/mL with epinephrine were slowly injected around the target nerve using US guidance and real-time assessment of the diffusion of the anesthetic solution. Ten minutes after the procedure the hiccups vanished and a partial sensomotoric block of his right shoulder developed. Our patient was discharged on the same day. In a post-care follow-up three days after discharge, our patient said that the shoulder block subsided in the evening of the very same day he was discharged home. He has not suffered from hiccups since. Permanent cure was confirmed by phone one month later and our patient approved of reporting the case.

## Discussion

One of the most frequent causes of self-limited hiccups is overdistension of the stomach, but more than 100 other causes of persistent and intractable hiccups have been described. Not all hiccups are benign, and so a possible underlying disease process must be kept in mind and examined when necessary [1]. In our case, one possible reason for persistent hiccups could be the genucubital position of the patient during the operation. This could cause extension of the neck, which in turn may have resulted in stretching the phrenic nerve roots. Traction on the diaphragm is also possible in the genucubital position.

As the persistent hiccups caused insomnia, exhaustion and made the discharge impossible and since both pharmacological and non-pharmacological treatments had proved ineffective, we ended up using a single-shot US-guided technique with our patient. The phrenic nerve could be demonstrated in his cervical region, despite its thinness, thanks to high-resolution ultrasonography and real-time imaging.

We used a fairly high dose of bupivacaine to ensure the blockade. This dose of bupivacaine actually resulted in a partial blockade of his right shoulder. A more caudal approach to the phrenic nerve and a smaller dose of the local anesthetic would probably have resulted in a lower spread of anesthesia. We considered the 5 mL dose of bupivacaine small enough to avoid its rostral spread to the root C4 [6], although even a smaller dose would probably have been sufficient, US being used to guide local anesthetic injection. Possible side effects also include difficulties in breathing as a consequence of unilateral diaphragmatic block. In this case we did not measure spirometry values in the way some authors have suggested [5], as our patient did not clinically present dyspneic or have a history of any lung disease. Furthermore, it has been shown that the incidence of diaphragmatic paralysis decreases and the respiratory function is preserved when a low volume of local anesthetic is used [7].

## Conclusions

As US offers non-invasive visual real-time information on the patient's anatomy and allows anesthesiologists to visualize needle insertion, to identify the exact location of the injected solution and to avoid such structures as arteries or veins, this method should be actively utilized. When both pharmacological and non-pharmacological treatments fail to cure persistent hiccups, a single-shot US-guided technique should be considered before the patient becomes exhausted. If a single-shot injection proves ineffective, continuous administration of local anesthetic via a catheter should be considered.

## Consent

Written informed consent was obtained from the patient for the publication of this case report. A copy of the written consent is available for review by the Editor-in-Chief of this journal.

## Competing interests

The authors declare that they have no competing interests.

## Authors' contributions

KK and VP wrote, revised, read and approved the final manuscript. VP performed the US-guided phrenic nerve block.
